# Part time patching treatment outcomes in children with amblyopia with and without fusion maldevelopment nystagmus: An eye movement study

**DOI:** 10.1371/journal.pone.0237346

**Published:** 2020-08-13

**Authors:** Matteo Scaramuzzi, Jordan Murray, Jorge Otero-Millan, Paolo Nucci, Aasef G. Shaikh, Fatema F. Ghasia

**Affiliations:** 1 Cole Eye Institute, Cleveland Clinic, Cleveland, OH, United States of America; 2 Department of Neuroscience, Unit of Ophthalmology, Istituto Giannina Gaslini, Genoa, Italy; 3 DISCCO, University of Milan, Milan, Italy; 4 Department of Neurology, The Johns Hopkins University, Baltimore, MD, United States of America; 5 Daroff—Dell’Osso Ocular Motility Laboratory, Cleveland, OH, United States of America; 6 Case Medical Center, Case Western Reserve University, Cleveland, OH, United States of America; Faculty of Medicine, Cairo University, EGYPT

## Abstract

**Purpose:**

We investigated how the abnormalities of fixation eye movements (FEMs) of the amblyopic eye were linked with treatment outcomes following part-time patching therapy in children with amblyopia.

**Methods:**

We recruited 53 patients, with at least 12 months of patching, and measured FEMs at the end of treatment. Subjects were classified based on FEM waveforms (those without nystagmus = 21, those with nystagmus without fusion maldevelopment nystagmus (FMN) = 21, and those with FMN = 11) and based on clinical type of amblyopia (anisometropic = 18, strabismic = 6, and mixed = 29). The treatment outcomes such as duration of treatment of receiving part-time patching therapy, visual acuity and stereo-acuity deficits at the end of treatment were determined. Bivariate contour ellipse area (BCEA), fast (fixational saccade/quick phases), and slow (inter-saccadic drifts/slow phases) FEMs of the fellow and amblyopic eye were analyzed.

**Results:**

Anisometropic group had less residual amblyopia (0.23±0.19logMAR acuity) compared to strabismic/mixed (0.36±0.26) groups (p = 0.007). Treatment duration in patients without nystagmus was lower (12.6±9.5months) compared to nystagmus without FMN (25.6±23.2) and FMN (29.5±20.4) groups (p = 0.006). Patients without nystagmus had better stereopsis at the end of treatment (2.3±0.84logarcsecs) compared to nystagmus without FMN (2.6±0.84) group (p = 0.003). The majority of patients with FMN (8/11) had absent stereopsis. BCEA of the amblyopic eye was higher in patients with greater residual visual acuity deficits in patients without nystagmus. No such association was seen in Nystagmus no FMN and FMN groups. Increased amplitude of fast FEMs, increased eye position variance and eye velocity of slow FEMs were seen in patients who had received longer duration of part time patching therapy and in those with greater residual amblyopia, and poor stereopsis at the end of treatment.

**Conclusions:**

Assessment of FEM waveforms and fast and slow FEM characteristics are important measures while describing fixation instability in amblyopia. Several FEM abnormalities were associated with stereo-acuity and visual acuity deficits and treatment duration in patients with amblyopia treated with part time patching therapy.

## Introduction

Amblyopia is a common neurodevelopmental disorder that affects 2–4% of the population. The most common treatment comprises of part-time patching therapy [1,2]. Despite good compliance, up to 40% of treated children have residual amblyopia [3].

Stable fixation is essential for clear vision; however, constant stimulation at a single foveal location can cause neural adaptation and visual fading. The physiologic fixational eye movements, namely microsaccades, drift, and tremor cause subtle changes in the foveal position of the target image and counteracts the fading [4,5]. Fixational saccades are thought to prevent visual fading, correct errors in fixation position arising from the drifts, and play an important role during everyday high visual acuity tasks [6–8]. The increase in fixation instability is detrimental to vision as shown by reduced visual acuity and reading speeds when simulating eye position instability [9,10]. Patients with amblyopia have an increase in fixation instability [11–17]. This instability could arise from the presence of nystagmus or due to increased amplitude of involuntary saccades and inter saccadic drifts elicited during fixation [18–20]. The majority of the studies quantify fixation instability using bivariate contour ellipse (BCEA) [17,21,22], which is a measure of the dispersion of eye position. However, BCEA does not parse the fast and slow eye movements [21], and it alone may underrepresent fixational instability arising from the nystagmus [20]. It also does not provide any information about the velocity of the eye–a metric that could affect treatment response [20,21,23].

Fusion Maldevelopment Nystagmus(FMN) is seen in patients who have experienced a disruption in binocularity in the first six months of life [24]. Animal models of amblyopia have suggested that the sensitive period arising from monocular deprivation in kittens are the first few months of life [25–28]. Experiments on non-human primates (NHP) with strabismus and amblyopia have revealed that loss of binocular connections within area V1(striate cortex) in the first months of life is the necessary and sufficient cause of FMN [29]. Tychsen and colleagues have shown that decorrelated visual experience can cause FMN and stereoacuity deficits in non-human primates with infantile strabismus, and the duration of binocular de-correlation is closely linked to the severity of FMN [14,24,30]. Although infantile strabismus is most commonly associated with FMN, any ocular pathology such as monocular deprivation and high anisometropia that causes binocular decorrelation in early infancy can produce FMN [24,28]. Early interventions such as strabismus or cataract surgery reduces the period of binocular decorrelation and has shown to produce good oculomotor outcomes [31–33].

During patching therapy, the amblyopic eye is the viewing eye which is known to have increased fixation instability^11^. In a previous study, we found that the presence of FMN was associated with a higher percentage of regression, required a longer duration of treatment, and had poor final stereopsis compared to patients with amblyopia without nystagmus.

[34]. Also, under monocular viewing conditions, the slow phase velocity of the un-occluded eye increases in patients with FMN which can affect treatment response [20,23,35–37]. We have found an increase in the amplitude of the fixational saccades and inter-saccadic drifts of the amblyopic eye, which correlate with the severity of visual acuity loss [19]. We hypothesize that the excessive motion of the target image on the fovea caused by abnormal fixation eye movements (FEMs) of the amblyopic eye would be associated with treatment outcomes. We evaluated the fast and slow FEMs of patients with amblyopia at the end of treatment as a function of three outcome measures, namely the visual acuity and stereo-acuity at the end of treatment and the treatment duration of part time patching therapy.

## Methods

We retrospectively reviewed the records of 80 patients with amblyopia from the practice of FG, who had eye movement recordings performed between 2013–2019. The Cleveland Clinic Institutional review board approved the experimental protocol and written informed consent was obtained from each participant’s parent/legal guardian in accordance with the Declaration of Helsinki. After review, 53 patients who had at least 12 months of follow up after initiation of part-time patching treatment were included (S1 Table). We have previously published a short report on these patients evaluating the association between the presence of FMN and the outcomes in patients with amblyopia treated with part time patching therapy [34].

The clinical categorization of amblyopia subtype (strabismic, anisometropic, mixed) and severity(mild, moderate, severe) at the time of diagnosis was based on PEDIG studies [38]. Type of amblyopia: Amblyopia associated with strabismus, anisometropia, or both meeting the following criteria: *1) Strabismic amblyopia*: At least one of the following criteria must be met, and criteria are not met for combined-mechanism amblyopia: a) Heterotropia at distance and/or near fixation on examination (with or without spectacles), b) History of strabismus surgery, c) Previous history of strabismus that has resolved with glasses and/ or surgery *2) Anisometropic amblyopia*: At least one of the following criteria must be met: a) ≥0.50 D difference between eyes in spherical equivalent or ≥1.50 D difference between eyes in astigmatism in any meridian; 3) *Mixed mechanism amblyopia*: Both of the following criteria must be met: a) criteria for strabismus are met (see above), b) ≥1.00 D difference between eyes in spherical equivalent or ≥1.50 D difference between eyes in astigmatism in any meridian. Severity of amblyopia: Mild amblyopia: if worse eye visual acuity (VA) was <0.30 LogMAR, moderate if ≥0.30 and <0.70, severe if ≥0.70.

Part-time patching (2–6 hours/day) was prescribed depending on the severity of amblyopia, 2 hours/ day in case of mild amblyopia (4 patients), 4 hours/day for moderate (30 patients) and 6 hours/day for severe (19 patients). Patients with manifest strabismus were treated according to the American Academy of Ophthalmology Preferred Practice Pattern [39]. In majority of the patients in the study, patching treatment was initiated prior to surgical treatment (S1 Table).

### Eye movement recording and analysis

Binocular horizontal and vertical eye positions were measured using a high-resolution video-based eye tracker (EyeLink 1000®, SR Research) [19,20], which has a spatial resolution of 0.01° and a temporal resolution of 500 Hz. Subjects wore their corrective lenses if applicable, while eye movements were recorded. Each subject's head was supported on a chin-rest, 55 cm away from the LCD screen. They fixated their gaze on a red filled circular visual target, with a diameter that subtended a 0.5° visual angle, on a white background (luminance 144 cd/m^2^) for 45 seconds. We used an infrared permissive filter that blocked the visible light but allowed eye movement measurements of the non-viewing eye. Monocular calibration and validation were performed per the manufacturer’s guidelines [19]. Eye movement recordings were obtained at the end of patching therapy.

FG evaluated the FEM traces obtained during both eye, fellow, and amblyopic eye viewing conditions and grouped the patients into those without and with nystagmus. Patients with nystagmus were further evaluated for the presence of FMN, defined as having a nasally directed slow phase under monocular viewing with the classic reversal in the direction of the quick phase towards the uncovered eye [40]. Patients with nystagmus who did not meet the criteria of FMN were categorized as having nystagmus without FMN. Patients with nystagmus without FMN did not have the dissociated vertical deviation frequently seen in patients with FMN. The form of the slow phases was decreasing or linear with dynamic overshoots of quick phases, unlike the increasing eye velocity waveforms seen in patients with infantile/congenital nystagmus. Blinks were identified and removed, as for the 200 milliseconds of data before and after them. Fixational saccades and quick phases of nystagmus were identified using an unsupervised clustering method [15,19,41]. The saccades amplitude is the absolute difference between eye positions at the start and end of a fixational saccade in patients without nystagmus or quick phase in patients with nystagmus. The composite amplitude of fixational saccades and quick phases was measured by computing the square root of the sum of the squared values of horizontal and vertical components. Drifts and slow phases were defined as epochs between fixational saccades and quick phases in patients without and with nystagmus respectively. We utilized horizontal and vertical eye positions independently to measure variance. The epochs of eye position signal between two adjacent fixational saccades (or quick phases) were used to compute the variance. To measure eye velocity, we differentiated the eye position signal using MATLAB^TM^ (MathWorks, Natick, MA). The velocity signal was smoothened with Savitzky-Golay filter, which can be applied to a set of digital data points for the smoothing purpose [19]. The composite eye velocity and eye position variance were computed for inter-saccadic drifts and slow phases (using the same formula as composite amplitude) in patients without and with nystagmus, respectively [20]. We also quantified the fixation stability by measuring a BCEA [42] and evaluated the treatment outcomes. All analyses were performed in MATLAB and GraphPad Prism 7 (La Jolla, USA).

### Clinical data and outcome measures

The age at diagnosis and follow up visits, visual acuity, strabismus measurements, stereopsis, and compliance to treatment were extracted from a retrospective chart review. Stereo-acuity was measured with the Titmus Stereoacuity Test (log arcsec). Patients with no stereo-acuity were assigned a value of 3.85 log arcsec. The total duration in months of patching treatment until visual acuity was stabilized with no further improvement or deterioration at ≥ 2 consecutive visits ≥ 6 weeks apart was computed for all the patients with at least 50% compliance. Investigators judged compliance with patching to be excellent (>75%—assigned a score of 4), good (51%–75%- assigned a score of 3), fair (26%–50%—assigned a score of 2), or poor (≤25%- assigned a score of 1), based on discussions with the family[43].

### Statistics

We described the clinical outcome measures (residual amblyopia expressed in logMAR acuity, treatment duration in months, and final stereopsis in logarc seconds at the end of treatment) per the amblyopia type and per the fixation eye movement characteristics separately. The Levene’s test of equality of error variances was employed and the test did not reach statistical significance across the clinical outcomes as a function of type and eye movement waveforms reported in the paper. We used one-way ANCOVA while controlling for age and severity of amblyopia at the time of diagnosis and compliance to treatment to assess treatment response. Bonferroni correction was employed for multiple pairwise comparisons. Pearson correlation coefficient was used to assess the relationship between frequencies of microsaccades and fixational saccades (amplitude <1° and >1°, respectively) and treatment outcomes. We also analyzed the fast (composite amplitude of fixational saccades/quick phases) and slow eye movement (eye position variance and eye velocities elicited during drifts and slow phase) parameters and BCEA of the amblyopic eye. For this analysis, the patients were divided based on residual amblyopia into those with logMAR acuity <0.30 and those with logMAR acuity ≥0.30. Patients were classified based on duration (months) of the treatment they underwent, as ≤6 months, 7–24 months and >24 months, and per the final stereopsis (log arcsec) at the end of the treatment into those with < 2 logarc sec, between 2–2.9 logarc sec and >2.9 logarc sec. We used one-way ANOVA with Bonferroni correction for statistically significant results. We report the values of Welch test when homogeneity of variance is violated. For comparisons between two groups, unpaired t-tests were used to assess treatment outcomes.

## Results

The study participants were grouped based on the type (anisometropic = 18, mixed = 29, strabismic = 6) and their fixation eye movement characteristics (No nystagmus = 21, Nystagmus no FMN = 21, FMN = 11). There was no difference in the age of patching treatment initiation, visual acuity at the start of treatment, and compliance to patching across clinical types, while the follow up of anisometropic patients was lower (Table 1).

**Table 1:** Baseline characteristics of subjects across clinical types.

	Anisometropic(n = 18)	Strabismic/Mixed(n = 35)	Mann-Whitney U Test p value
Age at Start of Patching Treatment (Months)	76.5 ± 14.5	63.5 ± 23.3	0.10
Visual Acuity at Start of Patching Treatment (LogMAR)	0.68±0.39	0.6 ± 0.35	0.64
Compliance to Patching	3.2±0.9	3 ± 1.1	0.18
Follow Up (Months)	46±31	66 ± 37	0.03

There was no difference in the age at start of patching, visual acuity at treatment initiation, compliance to patching and follow up across eye movement waveforms (Table 2).

**Table 2:** Baseline characteristics of subjects across eye movement waveforms.

	No Nystagmus (n = 21)	Nystagmus No FMN (n = 21)	FMN (n = 11)	One way ANOVA p value
Age at Start of Patching Treatment (Months)	69.6±20.0	74.7±23.6	65.5±35.9	0.60
Visual Acuity at Start of Patching Treatment (logMAR)	0.63±0.33	0.56±0.28	0.64±0.23	0.74
Compliance to Patching	2.8±1.2	3.1±1.2	2.8±0.87	0.57
Follow Up (Months)	56±34	71±37	75±43	0.32

### Clinical treatment outcomes

Patients with anisometropic amblyopia had less severe residual amblyopia compared to the strabismic/mixed groups [Anisometropia = 0.23±0.19, Strabismic/Mixed = 0.36±0.26 (ANCOVA F (1) = 7.8, p = 0.007, partial eta squared = 0.14)] while controlling for the age and severity of amblyopia and compliance to treatment. The severity of amblyopia at the start of treatment was associated with the presence of residual amblyopia (F(1) = 46.8, p = 0.0001, partial eta squared = 0.56). The treatment duration [Anisometropia = 15.5±17.04, Strabismic/Mixed = 24.5 ± 20.2 (F(1) = 2.1, p = 0.14)] and final stereopsis [Anisometropia = 2.5±0.92, Strabismic/Mixed = 2.7±0.92 (F(1) = 1.8, p = 0.17)] did not reach statistical significance while controlling for age and severity at start of treatment and compliance across the clinical types.

We also evaluated the clinical treatment outcomes as a function of fixation eye movement waveforms. There was no difference in residual amblyopia (logMAR acuity) [No nystagmus = 0.30±0.23,Nystagmus no FMN = 0.28±0.27 and FMN = 0.37±0.21(F(2) = 1.4, p = 0.25)] across the waveforms while controlling for age and severity of amblyopia at treatment initiation and compliance. The severity of amblyopia at the start of treatment was related with the extent of residual visual acuity deficit (F(1) = 41.1, p<0.001, partial eta squared = 0.46). The total duration of treatment (months) was higher in patients with nystagmus with and without FMN [No nystagmus = 12.6±9.5, Nystagmus no FMN = 25.6±23.2, FMN = 29.5±20.4 (F (2) = 5.7, p = 0.006, partial eta squared = 0.20)] while controlling for age and severity of amblyopia at treatment initiation and compliance. The severity of amblyopia at the start of treatment was associated with the treatment duration (F (1) = 6.2, p = 0.016, partial eta squared = 0.12). Post hoc pairwise comparisons revealed lower treatment duration between patients without nystagmus versus those with nystagmus no FMN (p = 0.013) and those with FMN (p = 0.04). No differences were seen between patients with nystagmus with and without FMN (p = 1.0). We also found that none of the patients without nystagmus received part time patching treatment > 2 years whereas none of the FMN patients received patching treatment for < 6 months. This is due to the plateau in response in patients without nystagmus resulting in discontinuation of therapy per clinical treatment guidelines. On the other hand FMN patients were at higher risk of regression, thus they received longer duration of patching therapy with duration > 6 months. The final stereopsis was better in patients without nystagmus [No nystagmus = 2.3±0.84, Nystagmus no FMN = 2.6±0.84 and FMN = 3.4±0.75 (F (2) = 6.5, p = 0.003, partial eta squared = 0.22)] while controlling for age and severity of amblyopia at treatment initiation and compliance. Post hoc pairwise comparisons revealed differences between patients without nystagmus versus those with FMN (p = 0.002) and between patients with nystagmus with and without FMN (p = 0.035). No differences were seen between patients without nystagmus and those with nystagmus without FMN (p = 0.907).

### Treatment outcomes and fixation eye movements

During patching therapy, the amblyopic eye is the viewing eye. Thus, we analyzed the eye movements of the amblyopic eye recorded under amblyopic eye viewing condition. We excluded patients who had missing eye movement data due to noise in horizontal and/or vertical eye position channels (n = 4, 2 = no nystagmus, 1 = nystagmus no FMN and 1 = FMN) from subsequent analysis.

### BCEA, nystagmus waveforms, and clinical outcomes

**Fig 1:**
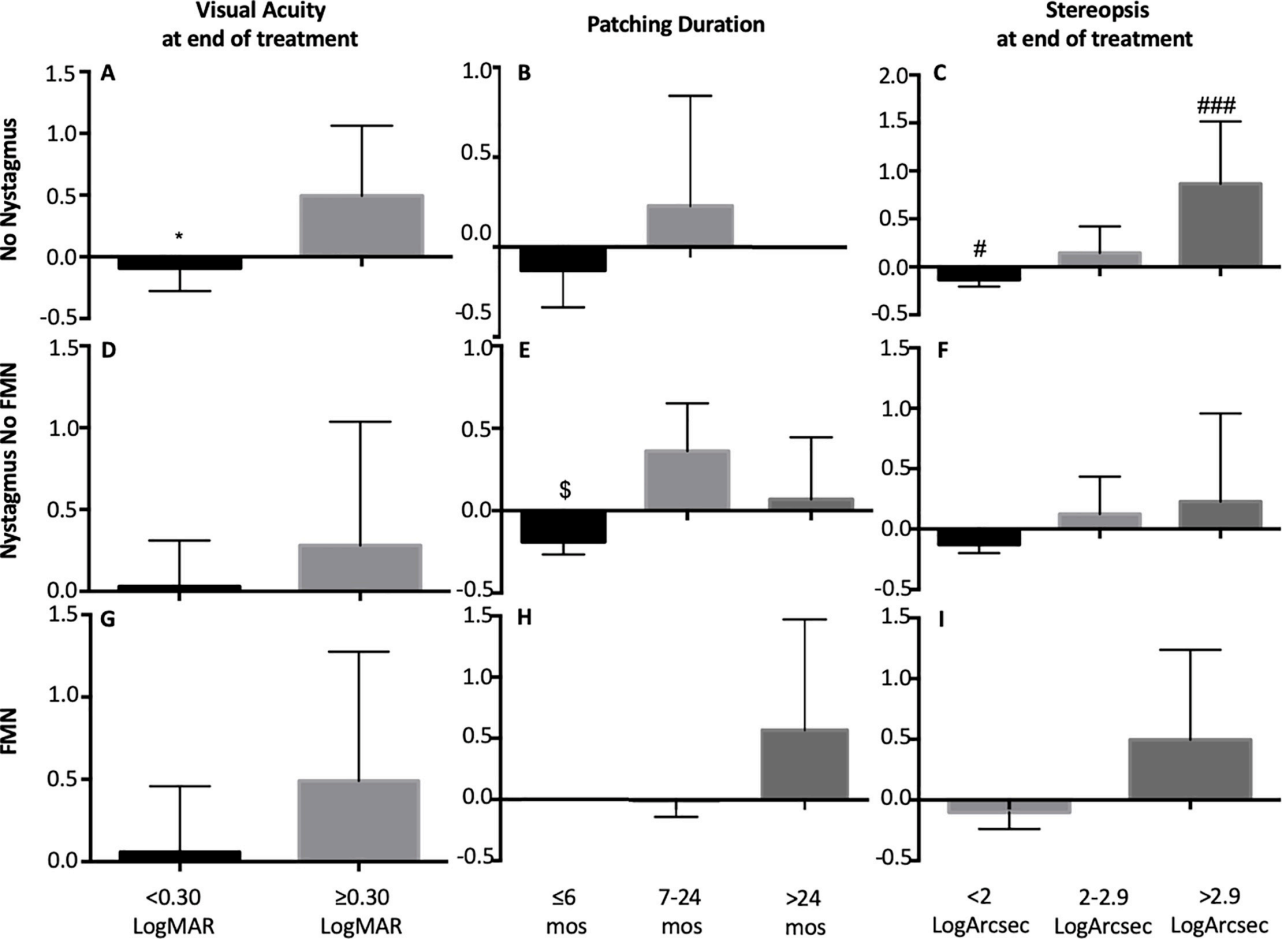
Mean and standard deviation of BCEA of the amblyopic eye as a function of treatment outcomes (Visual acuity at the end of treatment, Patching Duration and Stereopsis at the end of treatment) across FEM waveforms [no nystagmus- top (n = 19), nystagmus no FMN- middle (n = 19), FMN- bottom (n = 10). Unpaired t test was performed and statistical significance between the two groups is indicated by symbol * = < 0.3 logMAR vs. ≥0.30 logMAR. Post hoc bonferroni correction was performed and statistical significance between the groups is indicated by symbols: $ = ≤6 months vs. 7–24 months, # = < 2 log arc sec vs. 2–2.9 log arc sec, ### = < 2 log arc sec vs. > 2.9 log arc sec.

BCEA is a commonly employed global measure use to assess fixation stability. We found that the BCEA values of the fellow eye were better compared to the amblyopic eye. We have previously reported that BCEA value alone does not reflect the presence of nystagmus in amblyopia patients. In agreement with our previous studies, in this cohort, we found that BCEA values (log deg^2^) of the fellow eye [No nystagmus = -0.12 ± 0.39 (log deg^2^), Nystagmus no FMN = -0.07±0.33 (log deg^2^), FMN = -0.12 ± 0.35(log deg^2^), F (2) = 0.12, p = 0.88] and amblyopic eye [No nystagmus = 0.23±0.54 (log deg^2^), Nystagmus no FMN = 0.09±0.47(log deg^2^), FMN = 0.33±0.68(log deg^2^), F (2) = 0.753, p = 0.476] were similar in patients without nystagmus, patients with nystagmus without FMN and patients with FMN. We categorized the patients into three groups per their FEM waveforms and then analyzed the association between treatment outcomes and BCEA of the amblyopic eye within each subgroup (Fig 1).

In patients without nystagmus, we found that the BCEA of the amblyopic eye increased as a function of the severity of residual amblyopia and final stereopsis (Fig 1A and 1C). There was no correlation between BCEA of the amblyopic eye and treatment duration (Fig 1B). In patients with nystagmus without FMN, the BCEA of the amblyopic eye was lower in patients who received treatment for < 6 months (Fig 1E). There was no increase in the BCEA of the amblyopic eye as a function of residual amblyopia and stereopsis at the end of the treatment (Fig 1D and 1F). In patients with FMN, the BCEA of the amblyopic eye did not change as a function of the severity of residual amblyopia, treatment duration and final stereopsis (Fig 1G, 1H and 1I).

We have previously shown that the dynamic properties of slow and fast FEMs of both the fellow and amblyopic eye differ across the three fixation waveform groups [20]. In agreement with our previous study, we found that the fellow eye of patients with FMN had greater amplitude of quick phase compared to fixational saccade amplitude in no nystagmus and quick phases of nystagmus no FMN group [No nystagmus = 0.88 ± 1.1°, Nystagmus no FMN = 0.88 ± 0.55°, FMN = 1.2 ± 1.2°, F (2) = 32.6, p <0.0001]. We also found increased eye position variance and velocity of slow phases of the fellow eye in patients with FMN compared to the inter-saccadic drifts in patients without nystagmus and slow phase of nystagmus without FMN. [Eye velocity: No nystagmus = 0.71 ± 0.79°/s, Nystagmus no FMN = 0.69 ± 0.58°/s, FMN = 2.8 ± 3.5°/s, F (2) = 212.3, p <0.0001; Eye position variance: No nystagmus = 0.03 ± 0.05°, Nystagmus no FMN = 0.02 ± 0.04°, FMN = 0.05 ± 0.1°, F (2) = 34.3, p <0.0001]]. The amplitude of the fixational saccades in patients without nystagmus and amplitude of quick phases in patients with nystagmus were greater in the amblyopic eye compared to the fellow eye viewing. We found a similar result of increased eye velocity and eye position variance of the inter-saccadic drift in patients without nystagmus and slow phases of patients with nystagmus during amblyopic eye viewing compared to fellow eye viewing. During patching therapy, the amblyopic eye is the viewing eye. Thus, we have separately analyzed the treatment outcomes for the three groups per the FEM characteristics of the amblyopic eye (Table 3).

**Table 3:** Treatment outcomes as a function of the inter-saccadic drift velocities and eye position variance of the amblyopic eye across patients grouped by FEM waveform characteristics.

Visual Acuity at the End of treatment					
		< 0.30 logMAR		≥ 0.30 logMAR	t test
No nystagmus	Inter-saccadic drift velocities (°/s)	0.53 ± 0.40 (n = 9)	0.85 ± 0.83 (n = 10)		p<0.0001
	Eye position variance during inter-saccadic drifts (°)	0.02 ± 0.03 (n = 9)	0.06 ± 0.24 (n = 10)		p< 0.029
Nystagmus No FMN	Slow phase drift velocities (°/s)	0.93 ± 0.85 (n = 11)	0.98 ± 1.0 (n = 9)		p = 0.36
	Eye position variance during slow phases (°)	0.02 ± 0.12 (n = 11)	0.03 ± 0.87 (n = 9)		p = 0.23
FMN	Slow phase drift velocities (°/s)	1.2 ± 0.93 (n = 4)	7.5 ± 9.5 (n = 6)		p<0.0001
	Eye position variance during slow phases (°)	0.02 ± 0.06 (n = 4)	0.80 ± 2.1 (n = 6)		p<0.0001
Treatment Duration					
		≤6 months	7–24 months	> 24 months	ANOVA/ t test
No nystagmus	Inter-saccadic drift velocities(°/s)	0.48±0.42 (n = 8)	0.79±0.83 (n = 9)		p = 0.002
	Eye position variance during inter-saccadic drits (°)	0.023±0.03 (n = 8)	0.03±0.08 (n = 9)		p = 0.29
Nystagmus No FMN	Slow phase velocities (°/s) $, $ $, $ $ $	0.65±0.51 (n = 5)	1.3±1.1 (n = 6)	1.0±0.96 (n = 8)	F = 43.7, p<0.0001
	Eye position variance during slow phases (°) $, $ $ $	0.01±0.02 (n = 5)	0.04±0.09 (n = 6)	0.03±0.08 (n = 8)	F = 7.8, p<0.001
FMN	Slow phase velocities (°/s)		1.3±1.1(n = 5)	9.1±10.2 (n = 5)	p<0.0001
	Eye position variance during slow phases (°)		0.02±0.07(n = 5)	0.9±2.1(n = 5)	p<0.0001
Stereopsis at the end of treatment					
		< 2 log arc sec	2–2.9 log arc sec	> 2.0 log arc sec	ANOVA/t test
No nystagmus	Inter-saccadic drift velocities (°/s) ##, ###	0.63±0.52 (n = 7)	0.56±0.38 (n = 7)	0.92±0.99 (n = 5)	F = 4.0, p = 0.02
	Eye position variance (°) ###	0.02±0.03(n = 7)	0.03±0.04 (n = 7)	0.06±0.16(n = 5)	F = 3.6, p = 0.03
Nystagmus No FMN	Slow phase velocities (°/s) #, ##	0.70±0.62 (n = 4)	1.2±1.09 (n = 8)	0.70±0.59 (n = 8)	F = 36.0, p<0.001
	Eye position variance during slow phases (°) #, ##	0.01±0.02 (n = 4)	0.04±0.08 (n = 8)	0.02±0.05 (n = 8)	F = 11.7, p<0.001
FMN	Slow phase velocities (°/s)		1.2±0.95 (n = 3)	6.0±8.7 (n = 7)	p<0.0001
	Eye position variance during slow phases (°)		0.03±0.1(n = 3)	0.59±1.2 (n = 7)	p<0.0001

Post hoc bonferroni correction was performed and statistical significance between the groups is indicated by symbols

$ = ≤6 months vs. 7–24 months

$ $ = 7–24 months vs > 24months

$ $ $ = ≤6 months vs. > 24months

# = < 2 log arc sec vs. 2–2.9 log arc sec

## = 2–2.9 log arc sec vs. > 2.9 log arc sec

### = < 2 log arc sec vs. > 2.9 log arc sec

### Patients without nystagmus

**Fig 2:**
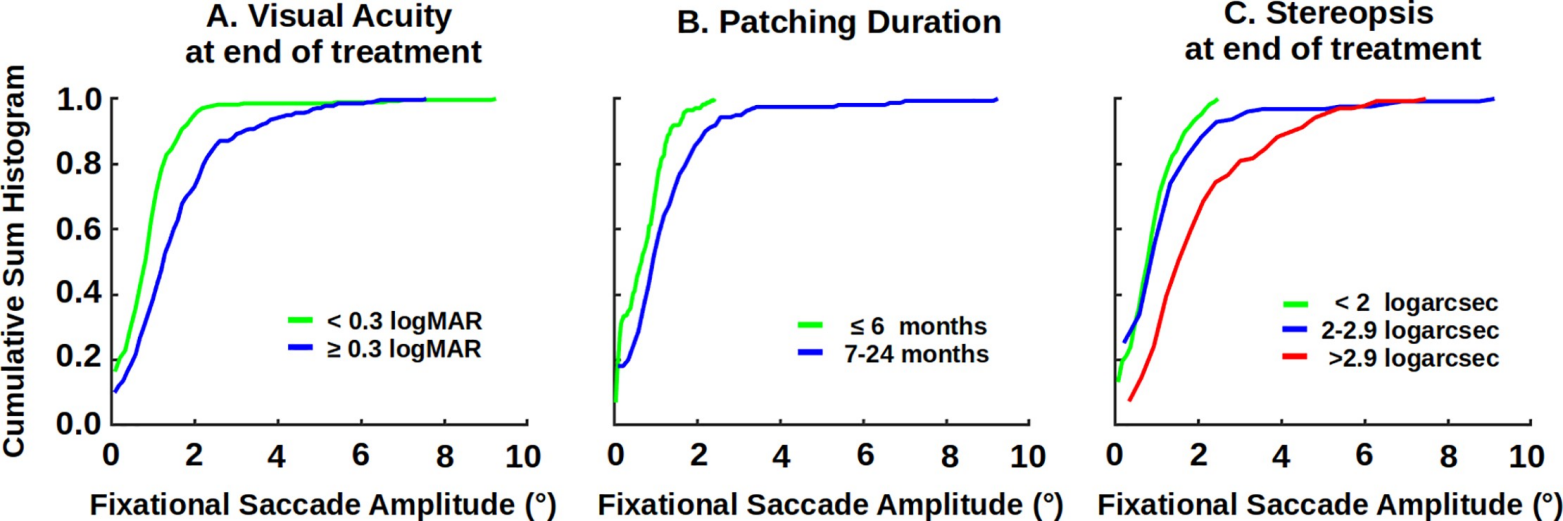
Cumulative sum histogram of fixational saccades of amblyopic eye of patients without nystagmus as a function of: A severity of residual amblyopia (green = <0.3 LogMAR (n = 9), blue = ≥0.3 LogMAR (n = 10)), B duration of treatment (green = duration ≤6 months (n = 8), blue = duration of 7–24 months (n = 9)), C stereopsis at the end of treatment (green (n = 6) = <2 LogArcsec, blue (n = 8) = 2–3 LogArcsec, and red (n = 5) = >3 LogArcsec).

These patients have increased fixational saccade amplitude and inter-saccadic drift of the amblyopic eye compared to controls [19]. Fig 2A demonstrates a rightward shift of the cumulative sum histogram of composite fixational saccade amplitude of the amblyopic eye as a function of residual amblyopia. There was an increase in the amplitude as a function of residual amblyopia [patients with <0.3 logMAR visual acuity = 0.93 ± 0.95 versus patients with ≥0.3 logMAR visual acuity = 1.5 ± 1.2; p<0.0001)]. Unlike the other two groups, none of the patients without nystagmus received >2 years of treatment. Patients treated for longer than six months had a greater amplitude of the fixational saccade of the amblyopic eye (Fig 2B) [≤6 months = 0.53 ± 0.51° and 7–24 months = 1.58 ± 2.04, t-test p<0.0001)]. This is indicated by the rightward shift of the distribution of the fixational saccades. We have previously reported increased fixational saccade amplitude in patients with poor stereopsis [19]. We found the amplitude of the fixational saccades related with final stereopsis (Fig 2C) [< 2 log arc sec = 0.86±0.6°; 2–2.9 log arc sec = 0.96±0.88°; > 2.9 log arc sec = 3.3±2.4°, F (2) = 44.7, p<0.0001)]. There were significant differences comparing patients with < 2 versus 2–2.9 log arc sec (p<0.0001), patients with 2–2.9 log arc sec versus >2.9 log arc sec (p<0.0001) and patients with < 2 vs >2.9 log arc sec (p<0.0001). We found a significant association between frequency of fixational saccades with amplitude >1° in patients with increasing severity of residual amblyopia (r = 0.59, p = 0.012, CI = 0.32–0.84) and those with worse stereopsis (r = 0.45,p = 0.06, CI = 0.03–0.87).

**Fig 3:**
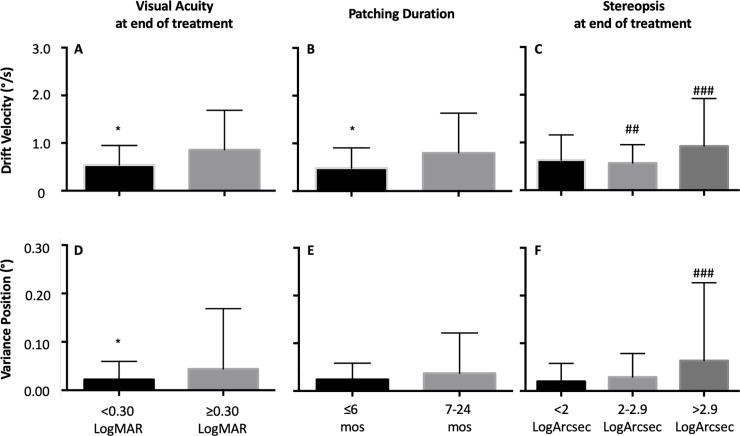
Mean and standard deviation of composite drift velocity and variance of eye position during inter-saccadic drift of the amblyopic eye of patients without nystagmus obtained under amblyopic eye viewing condition, as a function of severity of residual amblyopia (A and D [<0.3 LogMAR: n = 9; ≥0.3 LogMAR: n = 10), duration of treatment (B and E [≤6 months: n = 8; 7–24 months: n = 9]) and stereopsis at the end of treatment (C and F [<2 LogArcsec: n = 6; 2–3 LogArcsec: n = 8; >3 LogArcsec: n = 5]). Post hoc bonferroni correction was performed and statistical significance between the groups is indicated by symbols: # = <2 vs. 2–2.9 log arc sec, ## = 2–2.9 vs. >2.9 log arc sec, ### = <2 vs. >2.9 log arc sec.

The inter-saccadic drift velocities (Fig 3A) were lower in patients with less severe residual amblyopia. The drift velocities (Fig 3B) were higher in patients requiring intermediate duration of treatment. We found inter-saccadic drift velocities (Fig 3C) to be different as a function of final stereopsis. No significant difference comparing <2 versus 2–2.9 log arc sec (p = 1), while significant differences were present comparing <2 vs >2.9 log arc sec (p = 0.016) and 2–2.9 vs >2.9 log arc sec (p = 0.004). There was an increase in eye position variance as a function of severity of residual amblyopia (Fig 3D). No statistically significant differences were seen as a function of treatment duration (Fig 3E). We found a difference in eye position variance (Fig 3F) as a function of final stereopsis. Significant differences were seen between < 2 vs. >2.9 log arc sec (p = 0.001), whereas no differences were seen between < 2 versus 2–2.9 log arc sec (p = 1.0) and 2–2.9 vs > 2.9 log arc sec (p = 0.06) subgroups (Table 3).

The slow phase velocity is the pathology, while the quick phases are corrective that drives the development of nystagmus. Thus, we wanted to investigate whether there was a relation between clinical outcomes and the amplitude of quick phases, the eye velocity and position variance elicited during slow phases in patients with and without FMN.

### Patients with nystagmus no FMN

**Fig 4:**
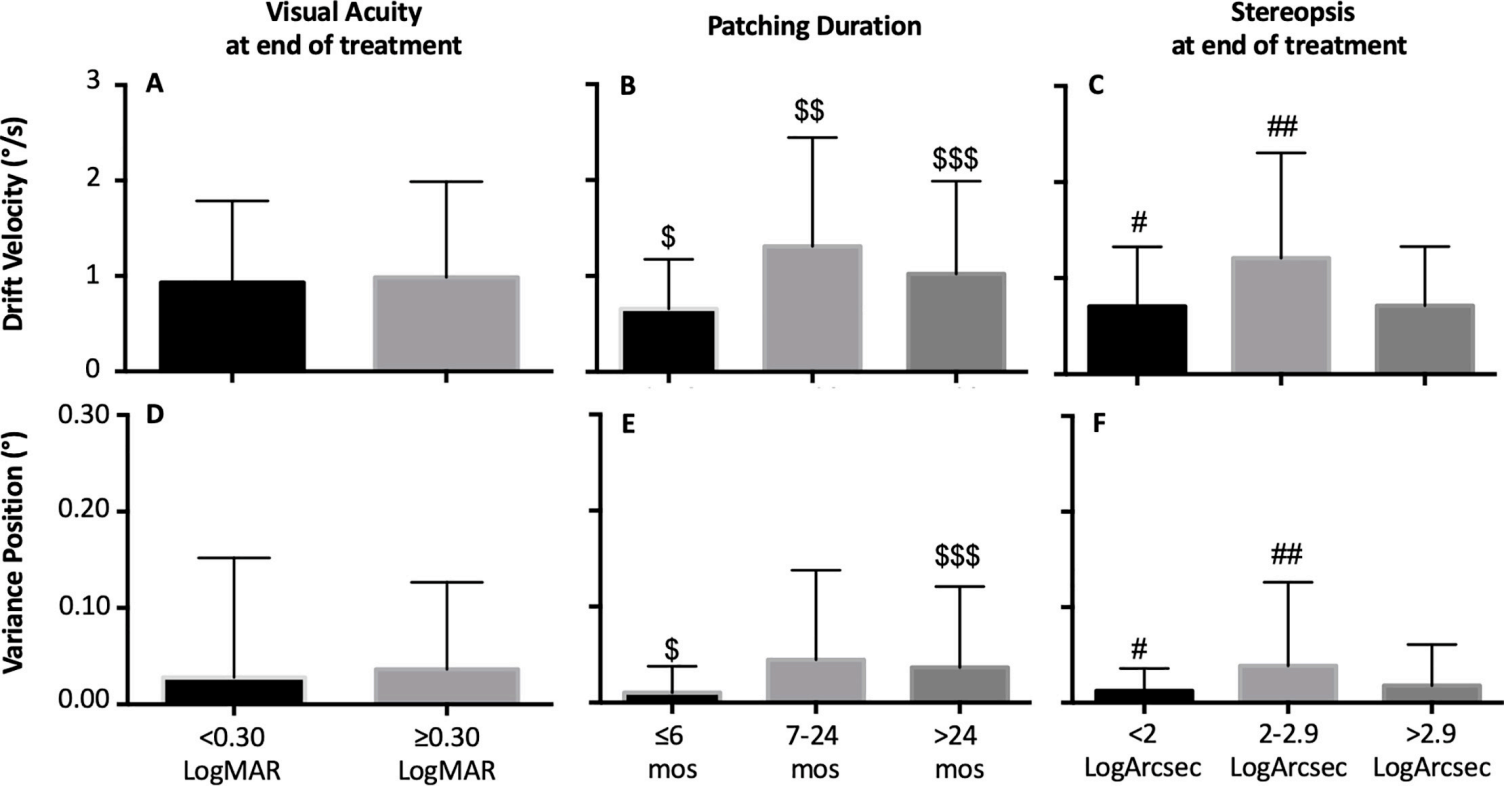
Mean and standard deviation of composite drift velocity and variance of eye position elicited during slow phase of the nystagmus in amblyopic eye of patients with nystagmus, without FMN, obtained under amblyopic eye viewing condition, as a function of severity of residual amblyopia (**A** and **D,** [<0.3 LogMAR: n = 11; ≥0.3 LogMAR: n = 9]), duration of treatment (**B** and **E** [≤6 months: n = 5; 7–24 months: n = 6; >24 months: n = 8]**)** and stereopsis at the end of treatment **(C** and **F** [<2 LogArcsec: n = 4; 2–2.9 LogArcsec: n = 8; >2.9 LogArcsec: n = 8]**)**. Post hoc bonferroni correction was performed and statistical significance between the groups is indicated by symbols: $ = ≤6 months vs. 7–24 months, $ $ = 7–24 months vs. >24 months, $ $ $ = ≤6 months vs. >24 months; # = <2 vs. 2–2.9 log arc sec, ## = 2–2.9 vs. >2.9 log arc sec, ### = <2 vs. >2.9 log arc sec.

We did not find any differences for eye velocities (Fig 4A) as a function of residual amblyopia. Similarly, we did not find any difference between eye position variance (Fig 4D) as a function of residual amblyopia. The eye velocities (Fig 4B) were higher in patients who received longer duration of treatment. There were significant differences between ≤6 months vs 7–24 months (p<0.0001), 7–24 months vs >24 months (p = 0.001) and ≤6 months vs. >24 months (p = 0.001). Similarly, the eye position variances were lower (Fig 4E) in patients that received shorter duration of treatment. Significant differences were present between ≤6 months vs 7–24 months (p<0.001), ≤6 months versus >24 months (p = 0.04) but no difference was seen between 7–24 months vs >24 months (p = 0.17) (Table 3).

The eye velocities (Fig 4C) were greater in patients with 2–2.9 log arc sec stereopsis compared to the other two groups. Significant differences were present comparing patients with stereopsis better than 2 log arc sec vs those with 2–2.9 log arc sec (p<0.0001) and patients with 2–2.9 log arc sec versus those with >2.9 log arc sec (p = 0.0001). No differences were seen between <2 log arc sec versus >2.9 log arc sec (p = 1.0). The eye position variances were greater in patients with 2–2.9 log arc sec compared to the other two groups (Fig 4F) as a function of final stereopsis. Significant differences were present comparing <2 vs 2–2.9 log arc sec (p = 0.005) and 2–2.9 vs >2.9 log arc sec (p = 0.003). No differences were seen between <2 versus >2.9 log arc sec (p = 1.0) (Table 3).

The amplitude of quick phases increased as a function of the severity of residual amblyopia [<0.3 LogMAR = 1.15±0.70°; ≥0.3 LogMAR = 1.6±1.3°; p<0.001)]. Similarly, the amplitude of the quick phase was related with treatment duration [≤6 months = 0.93°±0.49°; 7–24 months = 1.4±0.98°; >24 months = 1.2±0.73°; (F(2) = 44.0, p<0.0001)]. Significant differences were present comparing ≤6 vs 6–24 months (p<0.0001), 6–24 vs >24 months (p = 0.008) and ≤6 vs>24 months (p<0.0001). Also, the amplitude of the quick phase was linked with the final stereopsis [<2 log arc sec = 0.96±0.44°; 2–2.9 log arc sec = 1.3±0.86°; >2.9 log arc sec = 1.5±1.3°; (F(2) = 37.8, p<0.0001)]. There were significant differences comparing <2 vs 2–2.9 log arc sec (p<0.001) and 2–2.9 vs >2.9 log arc sec (p = 0.004) and <2 vs >2.9 log arc sec (p<0.001).

### Patients with FMN

**Fig 5:**
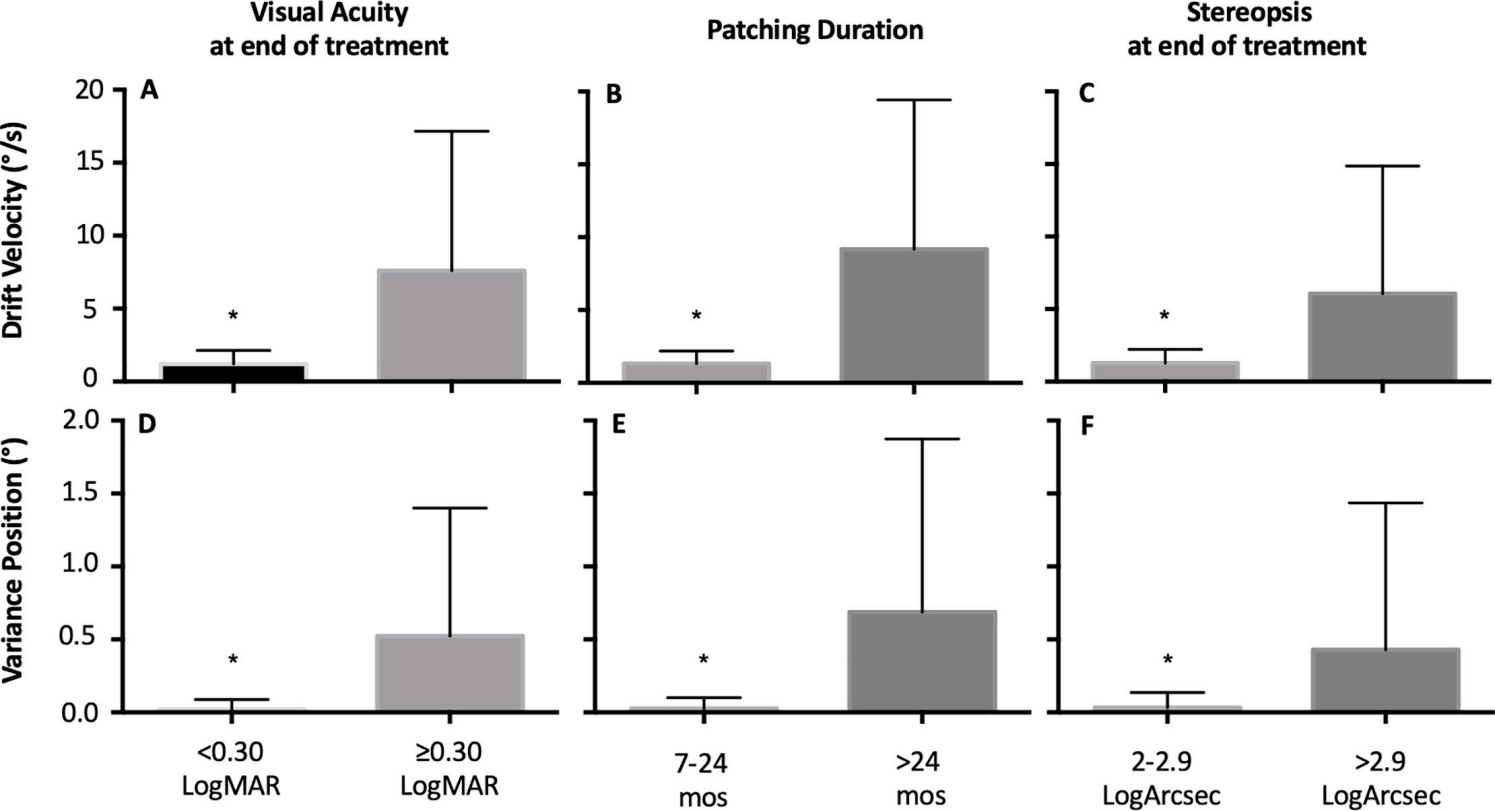
Mean and standard deviation of composite drift velocity and variance of eye position elicited during slow phase of the nystagmus in amblyopic eye of patients with FMN (n = 10), obtained under amblyopic eye viewing condition, as a function of severity of residual amblyopia (**A** and **D**[<0.3 LogMAR: n = 4; ≥0.3 LogMAR: n = 6]), duration of treatment (**B** and **E**[7–24 months: n = 6; >24 months: n = 4]**)** and stereopsis at the end of treatment **(C** and **F**[2–2.9 LogArcsec: n = 3; >2.9 LogArcsec: n = 7]**)**.

The slow phase velocities (Fig 5A) and the eye position variance (Fig 5D) were higher in patients with moderate residual amblyopia. Both the slow phase eye velocities (Fig 5B) and eye position variance (Fig 5E) were higher in patients that experienced long duration of treatment. Similarly, the slow phase velocities (Fig 5C) and the eye position variance (Fig 5F) were higher in patients with poor stereopsis (Table 3). The amplitude of quick phase was greater in patients with moderate residual amblyopia [<0.3 LogMAR = 1.2±1.2°vs ≥0.3 LogMAR = 1.5±1.02°; p<0.0001], treated for longer duration [7–24 months = 1.2±0.93° vs >24 months = 1.4±1.2°; p<0.0001] and in patients with poor stereopsis [2–2.9 log arc sec = 1.04±0.86° vs >2.9 log arc sec = 1.5±1.3°; p<0.0001].

## Discussion

We investigated the treatment outcomes in patients with amblyopia with and without fusion maldevelopment nystagmus and across clinical types. We found that patients without nystagmus had shorter duration of treatment with better stereopsis at the end of treatment. We also found that patients with FMN had improvement in visual acuity with part time patching therapy but required longer duration of treatment and have poor stereopsis at the end of treatment. We found that patients with amblyopia can have nystagmus without the reversal in the direction of the quick phase–a pre-requisite for diagnosis of FMN [44]. The presence of nystagmus without FMN was associated with longer durations of part time patching therapy but had better final stereopsis compared to FMN patients at the end of treatment. We found that anisometropic children with amblyopia had less severe residual amblyopia compared to strabismic and mixed amblyopia groups. The treatment duration and final stereopsis at the end of treatment were similar across the three clinical types of amblyopia while controlling for the age and severity of amblyopia at the start of treatment and compliance. In our cohort, we found that the severity of amblyopia at the start of treatment was related with the residual visual acuity deficits and duration of patching therapy. We did not find a similar correlation between final stereopsis at the end of treatment and severity of amblyopia at the start of treatment. This suggests that patients with more severe amblyopia are likely to have greater visual acuity deficits post treatment.

To the best of our knowledge, this is the first comprehensive study to evaluate the link between abnormalities of FEMs and the outcomes of part-time patching therapy in children affected by amblyopia with FMN. In our cohort, 27% of patients with strabismic/mixed amblyopia had FMN. Dell’Osso et al have reported a similar prevalence of FMN in strabismic patients [45]. We classified patients who did not meet the criteria of FMN and congenital motor nystagmus as nystagmus without FMN. Eye oscillations which are not FMN have been described by Birch in amblyopia patients [11]. The key features were the presence of nystagmus beats during viewing of either the amblyopic and/or fellow eye *without* the reversal in the direction of the nystagmus phase. The eye velocity waveforms were either linear of decelerating with presence of nystagmoid beats during the recordings. Few studies have investigated the outcomes of full-time patching therapy in patients with FMN. Simonsz et al have shown that the slow phase velocity of the amblyopic eye is greater during fixation of the fellow eye with a decrease in the velocity following complete occlusion of the fellow eye for several days. Thus, they have recommended full-time occlusion therapy [23]. Windsor has reported a reduction in amplitude, velocity, and frequency of quick phase of latent nystagmus in 3 patients by using cycloplegic eye drops in the amblyopic eye [46,47]. Von Noorden et al. have reported visual acuity improvement from 0–23.5 arcmin with a duration of treatment between 2–12 months in the patients with FMN (n = 12) following full-time occlusion therapy [36]. Eye movement recordings were not performed. Thus, they were not able to analyze the quick and slow phases of fusion maldevelopment nystagmus.

The presence of nystagmus and fast and slow fixation eye movement abnormalities, namely composite fast eye movement amplitude and eye velocity and eye position variance during slow fixational eye movements collectively contribute to fixation instability of the amblyopic eye [20,28]. Majority of the studies in the literature assess the stability of the fixation using BCEA measure. We found that the BCEA of the amblyopic and fellow eye are comparable across patients with and without nystagmus. In the current paper, we first categorized the patients with amblyopia based on their FEM waveforms and analyzed the BCEA values as a function of treatment outcomes. In patients without nystagmus, the BCEA of the amblyopic eye was higher in patients with greater visual acuity, and stereo acuity deficits post treatment, whereas, the BCEA values were similar in short versus intermediate duration of treatment. In patients with nystagmus without FMN, the BCEA of the amblyopic eye was lower in mild residual amblyopes and those treated for a shorter duration. In patients with FMN we found that the BCEA of the amblyopic eye was similar across the severity of residual visual acuity and stereoacuity deficits and treatment duration.

In the current manuscript, we parsed the FEM of the fellow and amblyopic eye and evaluated the fast and slow fixation components. We found that the amplitude, eye position variance and eye velocity of the fellow eye were greater compared to controls as reported in our previous studies [15,19,20]. These abnormalities were most pronounced in patients with FMN. We also examined the fast and slow FEMs of the amblyopic eye recorded at the end of treatment as a function of treatment outcomes. We found that in patients without nystagmus, those with greater residual amblyopia, poor final stereopsis, and those with prolonged treatment had increased frequency of fixational saccades >1deg, greater fixation saccade amplitude, and inter-saccadic drifts. In patients with FMN, the slow phase velocity and eye position variance and the amplitude of quick phase of the amblyopic eye were higher in patients with greater residual visual acuity and stereoacuity deficits and those that received a longer duration of treatment. It is possible that the increased slow phase velocity could arise due to prolonged duration of binocular decorrelation in early infancy too, as suggested by behavioral and neuroanatomic experiments in strabismic non- human primates [24], resulting in sub-optimal outcomes. Thus, the stereopsis deficits may be related to the timing of amblyopia development with the greatest impairment in patients with disruption of binocularity in infancy.

In patients with nystagmus without FMN, we did not find any differences for eye velocities and eye position variance of the slow phase of nystagmus as a function of residual amblyopia. The eye velocities and eye position variance during the slow phase of nystagmus were higher in patients that received longer duration of treatment. The eye velocities and eye variance of slow phase were greater in patients with some stereopsis compared to the other two groups. The presence of intermittent strabismus in patients with some stereopsis could have contributed to the high eye velocity and eye position variance of the amblyopic eye [15,18]. Similar to fixational saccades in patients without nystagmus, the amplitude of quick phases was higher in patients with greater residual visual acuity and stereoacuity deficits, and those that were treated for longer duration. Thus, this highlights the importance of evaluation of fixation eye movement waveforms and the assessment of slow and fast eye movement characteristics in patients with amblyopia.

Animal model studies have shown that the disruption of binocularity in the first few months of life is necessary and sufficient cause for the development of FMN. Thus, it is possible that the presence of anisometropia in early infancy is usually associated with the development of strabismus–resulting in mixed mechanism amblyopia. The low prevalence of pure anisometropic amblyopia in children <3 years old is consistent with this hypothesis [48]. We found that patients with anisometropic amblyopia were less likely to have FMN [20,34]. We compared the outcomes between anisometropic patients without nystagmus versus those with nystagmus without FMN. The subgroup analysis resulted in less number of subjects within each group however we found that anisometropic patients without nystagmus had less residual amblyopia, better stereopsis and less treatment duration compared to anisometropic patients with nystagmus without FMN. We did a similar subgroup analysis for patients with mixed and strabismic amblyopia as a function of fixation eye movement waveforms. Strabismic patients with and without FMN had similar residual visual acuity deficits. However, strabismic patients without nystagmus, had a shorter duration of treatment with better final stereopsis post treatment.

Fixational eye movements are increasingly recognized to play an essential role in vision and vision perception [5,19,34,49]. The abnormal FEMs cause excessive motion of the image on the fovea, which can have deleterious effects on vision [9,10,50]. Thus, it is possible that patients with greater abnormalities of fast and slow FEMs were more likely to have less favorable treatment outcomes with increased visual function deficits. Alternatively, the abnormal FEMs could be a consequence of vision loss. Nystagmus has been described in patients with acquired vision loss due to loss of optimum visual feedback from each eye [51,52]. Similar mechanisms could contribute to the increased inter saccadic drifts or nystagmus beats in patients with amblyopia- i.e. nystagmus without FMN could be a consequence of vision loss in early life.

A limitation of the current paper is that the eye movement recordings in the current study were obtained at the end of treatment, and the outcome measures were determined based on a retrospective chart review. This approach allowed us to include patients with a longer duration of follow up compared to most amblyopia treatment studies [38,43,53–58]. Thus, we were able to analyze occurrence of regression soon after the treatment was stopped or while it was being tapered. In the future, a prospective study of evaluating eye movement recordings obtained at the time of treatment initiation in a larger cohort of patients with longer duration of follow up than most of the current amblyopia treatment studies will allow us to analyze the effects of fixation waveforms within each clinical subtype and delineate the effects of presence of microstrabismus on treatment response. The eye movement abnormalities reported in the current paper could be a consequence of the severity of amblyopia at the beginning of treatment. Evaluation of the FEMs at the time of amblyopia diagnosis can provide insights into the factors that lead to development of nystagmus without FMN such as age at onset of amblyopia and severity of amblyopia. Thus, future prospective studies examining FEMs at the time of amblyopia diagnosis and how the FEMs change with treatment will be invaluable to understand the pathophysiology of various FEM abnormalities. If the fixation instability improves with treatment- it may serve as a useful marker to monitor treatment effectiveness as fixation instability has been shown to be associated with difficulties in everyday tasks such as reading and visual search [59,60].

Thus, to summarize, we examined the clinical outcomes following part-time occlusion therapy as a function of fixation eye movement waveforms and fast and slow eye movement parameters and fixation instability of the amblyopic eye. The analysis allowed us to examine the treatment response to patching therapy in patients with and without fusion maldevelopment nystagmus. The results highlight the importance of evaluating the waveforms for accurate diagnosis of FMN and analyzing the slow and fast eye movement characteristics rather than using BCEA alone as a measure of fixation instability. The data from the current study suggests that eye movement characterization and quantification can play an important role in amblyopia management. Similar to patching therapy, additional studies incorporating eye movement assessment should be designed to evaluate alternative treatments.

## Supporting information

S1 Table Demographic data of the enrolled subjects at the time of initiating patching treatment. (DOCX)

## References

[1] Holmes JM, Kraker RT, Beck RW, Birch EE, Cotter SA, Everett DF, et al A randomized trial of prescribed patching regimens for treatment of severe amblyopia in children. Ophthalmology. 2003 11;110(11):2075–87. 10.1016/j.ophtha.2003.08.001 14597512

[2] Wallace DK, Pediatric Eye Disease Investigator Group, Edwards AR, Cotter SA, Beck RW, Arnold RW, et al A randomized trial to evaluate 2 hours of daily patching for strabismic and anisometropic amblyopia in children. Ophthalmology. 2006 6;113(6):904–12. 10.1016/j.ophtha.2006.01.069 16751033 PMC1609192

[3] Scheiman MM. Randomized trial of treatment of amblyopia in children aged 7 to 17 years. Arch Ophthalmol. 2005 4;123(4):437–47. 10.1001/archopht.123.4.437 15824215

[4] Martinez-Conde S, Macknik SL, Troncoso XG, Hubel DH. Microsaccades: a neurophysiological analysis. Trends Neurosci [Internet]. 2009 9 [cited 2019 Oct 20];32(9):463–75. Available from: http://www.ncbi.nlm.nih.gov/pubmed/19716186 10.1016/j.tins.2009.05.006 19716186

[5] Martinez-Conde S, Macknik SL, Hubel DH. The role of fixational eye movements in visual perception. Vol. 5, Nature Reviews Neuroscience. Nature Publishing Group; 2004 p. 229–40. 10.1038/nrn1348 14976522

[6] Ko H-K, Poletti M, Rucci M. Microsaccades precisely relocate gaze in a high visual acuity task. Nat Neurosci [Internet]. 2010 12 [cited 2019 Nov 17];13(12):1549–53. Available from: http://www.ncbi.nlm.nih.gov/pubmed/21037583 10.1038/nn.2663 21037583 PMC3058801

[7] Troncoso XG, Macknik SL, Martinez-Conde S. Microsaccades counteract perceptual filling-in. J Vis [Internet]. 2008 11 4 [cited 2019 Nov 17];8(14):151–9. Available from: http://www.ncbi.nlm.nih.gov/pubmed/19146316 10.1167/8.14.15 19146316

[8] Costela FM, McCamy MB, Macknik SL, Otero-Millan J, Martinez-Conde S. Microsaccades restore the visibility of minute foveal targets. PeerJ [Internet]. 2013 [cited 2019 Nov 17];1:e119 Available from: http://www.ncbi.nlm.nih.gov/pubmed/23940832 10.7717/peerj.119 23940832 PMC3740150

[9] Falkenberg HK, Rubin GS, Bex PJ. Acuity, crowding, reading and fixation stability. Vision Res. 2007 1;47(1):126–35. 10.1016/j.visres.2006.09.014 17078991

[10] Macedo AF, Crossland MD, Rubin GS. The effect of retinal image slip on peripheral visual acuity. J Vis. 2008 11 12;8(14). 10.1167/8.14.16 19146317

[11] Birch EE. Amblyopia and binocular vision. Prog Retin Eye Res. 2013 3;33:67–84. 10.1016/j.preteyeres.2012.11.001 23201436 PMC3577063

[12] Chung STL, Kumar G, Li RW, Levi DM. Characteristics of fixational eye movements in amblyopia: Limitations on fixation stability and acuity? Vision Res. 2015 9 16;114:87–99. 10.1016/j.visres.2015.01.016 25668775 PMC4529398

[13] Shaikh AG, Ghasia FF. Fixational saccades are more disconjugate in adults than in children. PLoS One. 2017 4 13;12(4). 10.1371/journal.pone.0175295 PMC5391133 28406944

[14] Wong AMF. New concepts concerning the neural mechanisms of amblyopia and their clinical implications. Can J Ophthalmol [Internet]. 2012 10 [cited 2019 Nov 15];47(5):399–409. Available from: http://www.ncbi.nlm.nih.gov/pubmed/23036539 10.1016/j.jcjo.2012.05.002 23036539

[15] Ghasia FF, Otero-Millan J, Shaikh AG. Abnormal fixational eye movements in strabismus. Br J Ophthalmol. 2018 2 1;102(2):253–9. 10.1136/bjophthalmol-2017-310346 28698242

[16] Bedell HE, Yap YL, Flom MC. Fixational drift and nasal-temporal pursuit asymmetries in strabismic amblyopes. Invest Ophthalmol Vis Sci [Internet]. 1990 5 [cited 2020 Feb 5];31(5):968–76. Available from: http://www.ncbi.nlm.nih.gov/pubmed/2335458 2335458

[17] Subramanian V, Jost RM, Birch EE. A quantitative study of fixation stability in amblyopia. Invest Ophthalmol Vis Sci. 2013 3 19;54(3):1998–2003. 10.1167/iovs.12-11054 23372053 PMC3604910

[18] Pirdankar OH, Das VE. Influence of target parameters on fixation stability in normal and strabismic monkeys. Investig Ophthalmol Vis Sci. 2016 3 1;57(3):1087–95. 26968739 10.1167/iovs.15-17896 PMC4790473

[19] Shaikh AG, Otero-Millan J, Kumar P, Ghasia FF. Abnormal Fixational Eye Movements in Amblyopia. PLoS One. 2016 3 1;11(3). 10.1371/journal.pone.0149953 PMC4773232 26930079

[20] Kang SL, Beylergil SB, Otero-Millan J, Shaikh AG, Ghasia F. Fixational eye movement waveforms in amblyopia: Characteristics of fast and slow eye movements. J Eye Mov Res. 2019;in press. 10.16910/jemr.12.6.9 PMC7962684 33828757

[21] Castet E, Crossland M. Quantifying eye stability during a fixation task: a review of definitions and methods. Seeing Perceiving [Internet]. 2012 [cited 2019 Nov 17];25(5):449–69. Available from: http://www.ncbi.nlm.nih.gov/pubmed/22370759 10.1163/187847611X620955 22370759

[22] Ridder W, Patel R, Karsolia A, Duan D, Centeno L. Fixation Stability Before and After Amblyopia Therapy. [Internet]. Investigative Ophthalmology & Visual Science. 2019 p. 524 Available from: https://iovs.arvojournals.org/article.aspx?articleid = 2741195

[23] Simonsz HJ. The effect of prolonged monocular occlusion on latent nystagmus in the treatment of amblyopia. Doc Ophthalmol. 1989 8;72(3–4):375–84. 10.1007/BF00153506 2625098

[24] Tychsen L, Richards M, Wong A, Foeller P, Bradley D, Burkhalter A. The neural mechanism for Latent (fusion maldevelopment) nystagmus. J Neuroophthalmol. 2010 Sep;30(3):276–83. 10.1097/WNO.0b013e3181dfa9ca 20818206

[25] Hubel DH, Wiesel TN. The period of susceptibility to the physiological effects of unilateral eye closure in kittens. J Physiol. 1970 2 1;206(2):419–36. 10.1113/jphysiol.1970.sp009022 5498493 PMC1348655

[26] Wiesel TN, Hubel DH. Extent of recovery from the effects of visual deprivation in kittens. J Neurophysiol. 1965;28(6):1060–72. 10.1152/jn.1965.28.6.1060 5883732

[27] Crawford ML, Blake R, Cool SJ, von Noorden GK. Physiological consequences of unilateral and bilateral eye closure in macaque monkeys: some further observations. Brain Res [Internet]. 1975 1 24 [cited 2020 Feb 5];84(1):150–4. Available from: http://www.ncbi.nlm.nih.gov/pubmed/1111823 10.1016/0006-8993(75)90809-4 1111823

[28] Tusa RJ, Mustari MJ, Das VE, Boothe RG. Animal models for visual deprivation-induced strabismus and nystagmus In: Annals of the New York Academy of Sciences. New York Academy of Sciences; 2002 p. 346–60. 10.1111/j.1749-6632.2002.tb02833.x 11960818

[29] Tychsen L. Fusion Maldevelopment (Latent) Nystagmus: How Insights from Nonhuman Primate Experiments Have Benefitted Clinical Practice In: Advances in Translational Neuroscience of Eye Movement Disorders. Springer International Publishing; 2019 p. 255–70.

[30] Hasany A, Wong A, Foeller P, Bradley D, Tychsen L. Duration of binocular decorrelation in infancy predicts the severity of nasotemporal pursuit asymmetries in strabismic macaque monkeys. Neuroscience. 2008 10 2;156(2):403–11. 10.1016/j.neuroscience.2008.06.070 18708128 PMC2632802

[31] Lambert SR, Lynn MJ, Reeves R, Plager DA, Buckley EG, Wilson ME. Is there a latent period for the surgical treatment of children with dense bilateral congenital cataracts? J AAPOS Off Publ Am Assoc Pediatr Ophthalmol Strabismus. 2006;10(1):30–6. 10.1016/j.jaapos.2005.10.002 16527677

[32] Birch EE, Stager DR. Long-Term Motor and Sensory Outcomes After Early Surgery for Infantile Esotropia. J AAPOS. 2006 10;10(5):409–13. 10.1016/j.jaapos.2006.06.010 17070474

[33] Kelly KR, Cheng-Patel CS, Jost RM, Wang YZ, Birch EE. Fixation instability during binocular viewing in anisometropic and strabismic children. Exp Eye Res. 2019 6 1;183:29–37. 10.1016/j.exer.2018.07.013 30006273 PMC7323568

[34] Scaramuzzi M, Murray J, Otero-Millan J, Nucci P, Shaikh AG, Ghasia FF. Fixation instability in amblyopia: Oculomotor disease biomarkers predictive of treatment effectiveness In: Progress in Brain Research. Elsevier B.V.; 2019 p. 235–48. 10.1016/bs.pbr.2019.04.024 PMC8099169 31325983

[35] Abadi R V., Scallan CJ. Waveform characteristics of manifest latent nystagmus. Investig Ophthalmol Vis Sci. 2000;41(12):3805–17. 11053280

[36] von Noorden GK, Avilla C, Sidikaro Y, LaRoche R. Latent nystagmus and strabismic amblyopia. Am J Ophthalmol. 1987 1 15;103(1):87–9. 10.1016/s0002-9394(14)74174-1 3799793

[37] Duke-Elder S, Wybar KC. Ocular Motility and Strabismus. In: Mosby C V., editor. System of Ophthalmology. 1973 p. 824.

[38] Manh VM, Holmes JM, Lazar EL, Kraker RT, Wallace DK, Kulp MT, et al A Randomized Trial of a Binocular iPad Game Versus Part-Time Patching in Children Aged 13 to 16 Years With Amblyopia. Am J Ophthalmol. 2018 2;186:104–15. 10.1016/j.ajo.2017.11.017 29196184 PMC6206863

[39] American Academy of Ophthalmology. Pediatric Ophthalmology/Strabismus Summary Benchmarks [Internet]. 2019. Available from: https://www.aao.org/summary-benchmark-detail/pediatric-ophthalmology-strabismus-summary-benchma

[40] Dell’Osso LF, Schmidt D, Daroff RB. Latent, manifest latent, and congenital nystagmus. Arch Ophthalmol. 1979 10;97(10):1877–85. 10.1001/archopht.1979.01020020325008 485910

[41] Otero-Millan J, Castro JLA, Macknik SL, Martinez-Conde S. Unsupervised clustering method to detect microsaccades. J Vis. 2014 2 25;14(2):18–18. 10.1167/14.2.18 24569984

[42] Steinman RM, Cushman WB, Martins AJ. The precision of gaze. A review. Hum Neurobiol. 1982;1(2):97–109. 6764462

[43] Pediatric Eye Disease Investigator Group Writing Committee, Rutstein RP, Quinn GE, Lazar EL, Beck RW, Bonsall DJ, et al A randomized trial comparing Bangerter filters and patching for the treatment of moderate amblyopia in children. Ophthalmology. 2010;117(5):998–1004.e6. 10.1016/j.ophtha.2009.10.014 20163869 PMC2864338

[44] Hertle RW. A next step in naming and classification of eye movement disorders and strabismus. J AAPOS. 2002;6(4):201–2. 10.1067/mpa.2002.126491 12185342

[45] Dell’Osso LF. Congenital, latent and manifest latent nystagmus—similarities, differences and relation to strabismus. Jpn J Ophthalmol. 1985;29(4):351–68. 3831487

[46] Windsor CE, Burian HM, Milojevic B. Modification of Latent Nystagmus: Part I. Arch Ophthalmol. 1968;80(5):657–63. 10.1001/archopht.1968.00980050659014 5684313

[47] Windsor CE. Modification of Latent Nystagmus: Part II. Arch Ophthalmol. 1968;80(3):352 10.1001/archopht.1968.00980050354011 5670690

[48] Birch EE, Holmes JM. The clinical profile of amblyopia in children younger than 3 years of age. J AAPOS Off Publ Am Assoc Pediatr Ophthalmol Strabismus. 2010;14(6):494–7. 10.1016/j.jaapos.2010.10.004 PMC3310435 21168072

[49] Shi X-FF, Xu L-M, Li Y, Wang T, Zhao K-X, Sabel BA. Fixational saccadic eye movements are altered in anisometropic amblyopia. Restor Neurol Neurosci. 2012;30(6):445–62. 10.3233/RNN-2012-129000 23001901

[50] Castet E, Crossland M. Quantifying eye stability during a fixation task: A review of definitions and methods. Seeing Perceiving. 2012;25(5):449–69. 10.1163/187847611X620955 22370759

[51] Leigh JR, Zee DS. The Neurology of Eye Movements (5 ed.). Vol. 8, Neuropsychologia. Oxford University Press; 2019. 1–35 p.

[52] Schneider RM, Thurtell MJ, Eisele S, Lincoff N, Bala E, Leigh RJ. Neurological Basis for Eye Movements of the Blind. PLoS One. 2013;8(2):e56556 10.1371/journal.pone.0056556 23441203 PMC3575504

[53] Holmes JM, Manh VM, Lazar EL, Beck RW, Birch EE, Kraker RT, et al Effect of a Binocular iPad Game vs Part-time Patching in Children Aged 5 to 12 Years With Amblyopia: A Randomized Clinical Trial. JAMA Ophthalmol. 2016;134(12):1391–400. 10.1001/jamaophthalmol.2016.4262 27812703 PMC5145771

[54] Pediatric Eye Disease Investigator Group (PEDIG). A Randomized Trial of Atropine vs Patching for Treatment of Moderate Amblyopia in Children | Ophthalmology | JAMA Ophthalmology | JAMA Network. 2002;268–78. 10.1001/archopht.120.3.268 11879129

[55] Pediatric Eye Disease Investigator Group, Chen AM, Holmes JM, Chandler DL, Patel RA, Gray ME, et al A Randomized Trial Evaluating Short-term Effectiveness of Overminus Lenses in Children 3 to 6 Years of Age with Intermittent Exotropia. Ophthalmology. 2016;123(10):2127–36. 10.1016/j.ophtha.2016.06.042 27506485 PMC5035588

[56] Pediatric Eye Disease Investigator Group, Mohney BG, Cotter SA, Chandler DL, Holmes JM, Chen AM, et al A Randomized Trial Comparing Part-time Patching with Observation for Intermittent Exotropia in Children 12 to 35 Months of Age. Ophthalmology. 2015;122(8):1718–25. 10.1016/j.ophtha.2015.04.025 26072346 PMC4516562

[57] Pediatric Eye Disease Investigator Group, Cotter SA, Mohney BG, Chandler DL, Holmes JM, Repka MX, et al A randomized trial comparing part-time patching with observation for children 3 to 10 years of age with intermittent exotropia. Ophthalmology. 2014;121(12):2299–310. 10.1016/j.ophtha.2014.07.021 25234012 PMC4253733

[58] Repka MX, Kraker RT, Beck RW, Birch E, Cotter SA, Holmes JM, et al Treatment of severe amblyopia with weekend atropine: Results from 2 randomized clinical trials. J AAPOS. 2009 Jun;13(3):258–63. 10.1016/j.jaapos.2009.03.002 19541265 PMC2713117

[59] Kelly KR, Jost RM, De La Cruz A, Birch EE. Amblyopic children read more slowly than controls under natural, binocular reading conditions Presented at the 41st Annual Meeting of the American Association for Pediatric Ophthalmology and Strabismus, New Orleans, Louisiana, March 25–29, 2015. In: Journal of AAPOS. Mosby Inc.; 2015 p. 515–20. 10.1016/j.jaapos.2015.09.002 PMC4688187 26610788

[60] Chen D, Otero-Millan J, Kumar P, Shaikh AG, Ghasia FF. Visual Search in Amblyopia: Abnormal Fixational Eye Movements and Suboptimal Sampling Strategies. Invest Ophthalmol Vis Sci. 2018;59(11):4506–17. 10.1167/iovs.18-24794 30208418

