# Supplementary material for: Part time patching treatment outcomes in children with amblyopia with and without fusion maldevelopment nystagmus: An eye movement study
Source: PLoS One. 2020 Aug 13;15(8):e0237346. doi: 10.1371/journal.pone.0237346 (PMC7425965; doi:10.1371/journal.pone.0237346)
Supplement: S1 Table — (DOCX) [file pone.0237346.s001.docx]

**S1 Table.**

**Demographic data of the enrolled subjects at the time of initiating patching treatment.**

| Gender | Age  (Months) | Type  And  Severity | Visual Acuity  (LogMAR) | Stereo  acuity  (arc second) | Eye Movement Waveform | Ref  OD | Ref  OS | Strabismus Near (cc) | Strabismus Distance (cc) | Surgery |
| --- | --- | --- | --- | --- | --- | --- | --- | --- | --- | --- |
| F | 65 | Strabismic  Severe | 0.2  0.7 | 7000 | None | +3.5  +0.75 x 120 | +3.75  +0.75 x 60 | Ortho cc | Ortho cc | None |
| F | 27 | Strabismic  Severe | 1.4  0.3 | 7000 | None | +6.5 | +6.25 | ET 30 | ET 30 | BMR REC  Age 36 months |
| M | 69 | Mixed  Moderate | 0.5  0 | 200 | None | +5 | +1 | ET 35 | ET 35 | R&R OD  Age 72 months |
| F | 82 | Mixed  Moderate | 0.4  0.1 | 100 | None | +3.0  +1.25 x 65 | +1.25  +0.25 x 115 | ET 12 | E(T) 4-6 | None |
| F | 71 | Strabismic  Moderate | 1.0  0.3 | 7000 | None | +4 | +4 | 6-8 E(T) | Ortho cc | None |
| F | 57 | Mixed  Moderate | 0.3  0 | 40 | None | +8.50  +1.75 x 70 | +7.5  +1.5 x 110 | Ortho cc | Ortho cc | None |
| M | 44 | Mixed  Moderate | 0.2  0.5 | 140 | None | +2.5 | +4.5 | Ortho cc | Ortho cc | None |
| M | 84 | Mixed Moderate | 0  0.4 | 100 | None | Plano  +0.50 x 95 | -0.75  +3.5 x 85 | LX(T) 12 | LX(T) 14 | None |
| M | 90 | Anisometropic  Moderate | 0.2  0.5 | 50 | None | Plano  +0.50 x 85 | +5.25  +2.00 x 105 | Ortho | Ortho | None |
| M | 90 | Anisometropic  Severe | 1.2  0 | 140 | None | +7.00  +0.50 x 60 | +1.00  +0.25 x 50 | Ortho | Ortho | None |
| M | 46 | Mixed  Severe | 0  0.8 | 140 | None | +6.5  2.00 x 70 | +0.5  +0.5 x 90 | Ortho cc | Ortho cc | None |
| M | 79 | Anisometropic  Moderate | 0  0.5 | 7000 | None | Plano  +0.75 x 95 | +4.25  +2.00 x 90 | Ortho | Ortho | None |
| F | 81 | Anisometropic  Moderate | 0  0.6 | 140 | None | +0.25  +0.25 x 90 | +5  +0.5 x 100 | Ortho | Ortho | None |
| F | 66 | Anisometropic  Moderate | 0.5  0.1 | 80 | None | +5  +0.50x100 | +3  +0.50 x 80 | Ortho | Ortho | None |
| F | 60 | Anisometropic  Severe | 0.7  0.1 | 50 | None | +7.5 | +5.0  +0.50 x 180 | Ortho | Ortho | None |
| M | 53 | Anisometropic  Moderate | 0.55  0.2 | 40 | None | +4  +0.50x 105 | +0.5  +0.5 x 85 | Ortho | Ortho | None |
| F | 117 | Anisometropic  Mild | 0  0.2 | 40 | None | -0.25  +0.5 x 90 | Plano  +2 x 85 | Ortho | Ortho | None |
| F | 81 | Anisometropic  Moderate | 0.4  0 | 60 | None | -2.75  +4.25 x 95 | +1.5 | Ortho | Ortho | None |
| F | 53 | Anisometropic  Moderate | 0.1  0.6 | 60 | None | +0.5  +1.00 x 90 | +3.5  +1.00 x 90 | Ortho | Ortho | None |
| M | 60 | Mixed  Severe | 0.8  0.1 | 7000 | None | +5.25  +2.00 x 75 | -0.5  +0.5 x 95 | ET 10 | ET 10 | None |
| F | 87 | Anisometropic  Severe | 1.2  0.3 | 100 | None | -12  +1 x 105 | -0.25  +1.25 x 75 | Ortho | Ortho | None |
| F | 63 | Anisometropic  Moderate | 0.4  0 | 40 | Nystagmus No FMN | +4.25  +1.0 x 95 | +1.75  +0.25 x 80 | Ortho | Ortho | None |
| M | 63 | Anisometropic  Severe | 0.1  1.9 | 140 | Nystagmus No FMN | +0.25  +0.5 x 90 | -10.75  + 2.0 x 50 | Ortho | Ortho | None |
| M | 80 | Anisometropic  Moderate | 0.2  0.4 | 100 | Nystagmus No FMN | +7.25  +1.5 x 90 | +8.25  +1.5 x 100 | Ortho | Ortho | None |
| F | 83 | Mixed  Moderate | 0.2  0.6 | 100 | Nystagmus No FMN | -1.75  +3 x 85 | -10.00  +3.75 x 85 | Ortho cc | Ortho cc | None |
| M | 75 | Anisometropic  Severe | 0.8  0 | 200 | Nystagmus No FMN | +6.75  +3 x 90 | +0.5 | Ortho | Ortho | None |
| F | 85 | Anisometropic  Severe | 0  0.8 | 3500 | Nystagmus No FMN | -0.25 | -12.5  +3.5 x 120 | Ortho | Ortho | None |
| M | 48 | Mixed  Severe | 1.0  0 | 7000 | Nystagmus No FMN | +4.5  +1.00 x 60 | +1.5  +0.50 x 120 | 10 RE(T) | 8 RE(T) | None |
| F | 71 | Anisometropic  Moderate | 0.4  0 | 100 | Nystagmus No FMN | +4  +1.25 x 85 | +1.5  +0.5 x 85 | Ortho | Ortho | None |
| F | 66 | Mixed  Severe | 0.3  0.7 | 7000 | Nystagmus No FMN | +2.25  +0.75 x 80 | +3.5  +0.5 x 135 | Ortho cc | Ortho cc | None |
| M | 63 | Mixed  Mild | 0.2  0 | 7000 | Nystagmus No FMN | +1.25  +0.75 x110 | +0.25  +2.0 x 80 | Ortho cc | Ortho cc | None |
| F | 95 | Mixed  Moderate | 0.4  0.1 | 200 | Nystagmus No FMN | -11.5  +0.75 x 75 | -6.5  +1.0 x 105 | XT 20 | XT 25 | REC RL OD  Age 84 months |
| M | 102 | Mixed  Mild | 0.1  0.2 | 100 | Nystagmus No FMN | +6  +2.0 x 90 | +7  +1.75 x 90 | Ortho cc | Ortho cc | None |
| M | 33 | Mixed  Severe | 0.2  0.7 | 3500 | Nystagmus No FMN | +1.50 | +4 | LE(T) 8 | LE(T) 10 | None |
| F | 41 | Mixed  Moderate | 0.1  0.3 | 50 | Nystagmus No FMN | +5.5  +1.00 x100 | +6.5  +1.0 x 80 | Ortho cc | Ortho cc | None |
| F | 77 | Mixed  Moderate | 0  0.3 | 7000 | Nystagmus No FMN | +4.50  +2.00 x 90 | +5.5  +2.25 x 90 | E(T) 8 | E(T) 10 | None |
| M | 39 | Strabismic  Moderate | 0  0.3 | 140 | Nystagmus No FMN | +2.75  +0.50 x180 | +2.75  +0.50 x 180 | ET 35 | ET 35 | BMR REC BIOmyectomy at age 36 months |
| F | 81 | Anisometropic  Moderate | 0  0.5 | 80 | Nystagmus No FMN | +1.00  +0.5 x 90 | +3.75 | Ortho | Ortho | None |
| F | 12 | Mixed  Severe | 0.4  0.8 | 7000 | Nystagmus No FMN | +1.50  +0.50 x 70 | +3.5  +0.5 x 120 | E(T) 8 | E(T) 8 | None |
| F | 82 | Mixed  Mild | 0  0.2 | 80 | Nystagmus No FMN | -1.5  +0.75 x 90 | -2.5  +1.00 x 90 | XT 35 | XT 35 | BLR REC BIOmyectomy  Age 96 |
| F | 84 | Mixed  Moderate | 0.1  0.4 | 200 | Nystagmus No FMN | -0.75  +0.5 x 75 | +1.5  +1.00 x 90 | XT 25 | XT 30 | REC RL OS  Age 84 months |
| M | 131 | Mixed  Moderate | 0.5  0.2 | 7000 | Nystagmus No FMN | +3.50  +0.50 x110 | +1.00  +0.50 x 90 | 50 RET | 50 RET | R&R  Age 144 months |
| M | 72 | Mixed  Severe | 0.2  0.7 | 7000 | FMN | +5.00  +0.50 x 90 | +6.25  +1.00 x 95 | Ortho cc | Ortho cc | None |
| F | 14 | Strabismic  Severe | 0.7  0.2 | 7000 | FMN | +3.50  +1.75 x 90 | +3.50  +1.75 x 90 | RE(T) 8-10 | RE(T) 10 | BMR REC  Age 12 months |
| M | 80 | Mixed  Moderate | 0.55  0 | 7000 | FMN | -9.5  +2.5 x 165 | plano  +0.75 x 45 | ET 4 | ET 4 | None |
| M | 66 | Strabismic  Severe | 1.0  0.2 | 7000 | FMN | +3 | +3 | XT >60 | XT >60 | BLR REC  Age 18 months |
| F | 67 | Mixed  Moderate | 0  0.6 | 7000 | FMN | +5  +1.5 x 80 | +6  +1.5 x 95 | Ortho cc | Ortho cc | None |
| M | 83 | Mixed  Severe | 0.4  0.8 | 7000 | FMN | -6.75  +3.75 x 90 | -9.0  +3.75 x 90 | XT 25 | XT 45 | BMR REC  Age 96 months |
| M | 18 | Mixed  Moderate | 0.3  0.2 | 7000 | FMN | +4.5 | +3.5 | ET 30 | ET 25 | BMR REC  Age 36 months |
| M | 86 | Mixed  Severe | 1.0  0.4 | 7000 | FMN | +4.5  +2.75 x 85 | +3.5  +2.75 x 95 | XT 12 | XT 12 | None |
| M | 85 | Mixed  Moderate | 0  0.6 | 80 | FMN | -0.5  +1.00 x110 | +2.50  +1.50 x 55 | Flick XT | Flick XT | None |
| F | 60 | Mixed Moderate | 0.3  0.1 | 400 | FMN | +4 | +2.25 | XT 20  LHT 8 | XT 20  LHT 8 | BLR REC  BIO myectomy age 60 months |
| M | 30 | Mixed  Moderate | 0.5  0.3 | 200 | FMN | +8.00  +1.5 x 90 | +7.25  +0.5 x 90 | ET 6-8 | ET 4 | None |

BLR: Bilateral Lateral Recti Muscles. BMR: Bilateral Medial Recti Muscles. CC: with correction. ET: Esotropia. F: Female. LE: Left Eye. M: Male. RE: Right Eye. REC: Recession. R&R: Recession and Resection. XT: Exotropia
